# Double-scope technique to recover from hand-suturing trouble in the duodenum

**DOI:** 10.1055/a-2199-3398

**Published:** 2023-11-20

**Authors:** Leonardo Yoshio Sato, Yoshitaka Hata, Mitsuru Esaki, Eikichi Ihara, Shiho Tajiri, Tomohiko Moriyama, Yosuke Minoda

**Affiliations:** 1 Department of Endoscopy (Endobatel), Hospital Vita Batel, Curitiba, Brazil; 2 145181 International Medical Department, Kyushu University Hospital, Fukuoka, Japan; 3 Department of Medicine and Bioregulatory Science, Kyushu University Graduate School of Medical Sciences, Fukuoka, Japan; 4 145181 Department of Endoscopic Diagnostics Therapeutics, Kyushu University Hospital, Fukuoka, Japan

Duodenal adenomas are uncommon, with a prevalence of 0.3%–4.6% 1 2 . Endoscopic resection (ER) is the standard treatment for a solitary adenoma. As ER for duodenal lesions is associated with a high risk of adverse events, such as bleeding and delayed perforation, closure of post-ER ulcers has been reported as being effective in reducing the risk of complications 3 4 5 .

Herein, we report the case of a 52-year-old woman who underwent endoscopic mucosal resection (EMR) for a 5-mm lesion in the second portion of the duodenum. This lesion had been observed during a previous duodenoscopy as part of a medical check-up ( Fig. 1 a). The EMR procedure was successfully completed using a snare. Covering or closing post-EMR ulcers is an effective strategy to reduce the risk of complications. Therefore, a closure procedure was performed using a needle attached to a suture line and an endoscopic hand-suturing device ( Fig. 1 b,c). The mucosal defect was completely closed by suturing; however, the thread-cutter device became stuck on the suture and could not be opened while removing the excess line ( Fig. 1 d). Given this situation, a second ultrathin endoscope with a 2.4-mm instrument channel was inserted to address the issue ( Fig. 1 e). The endoscopic view provided by the second scope enabled better decision-making about the subsequent treatment. An electrical knife was inserted through the second endoscope to cut the suture (spray mode, 40 W, effect 1) ( Fig. 1 f,g). The procedure was completed without any additional complications ( Fig. 1 h), leading to an ultimately successful treatment ( Video 1 ).

The double-scope technique requires the use of an ultrathin endoscope, the recent development of which has enabled their use in various techniques. This method proved to be beneficial in managing the critical situation in this case. When employing the double-scope technique for duodenal lesions, it is crucial to adequately lubricate the second scope. This ensures that it can pass smoothly through the pylorus without obstruction from the first scope.

**Fig. 1:**
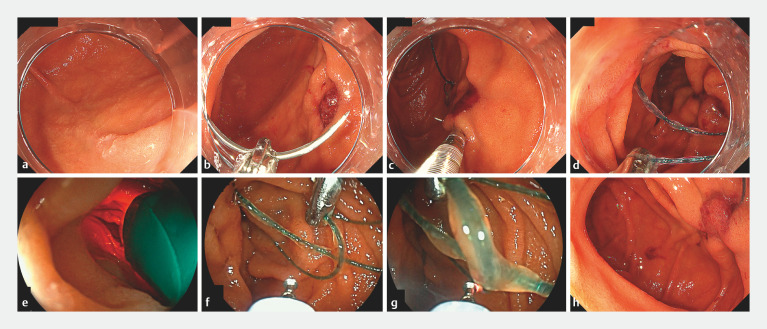
Endoscopic images of the double-scope technique being used to recover from hand-suturing trouble in the duodenum showing; **a** a 5-mm lesion in the second portion of the duodenum; **b** the post-endoscopic mucosal resection ulcer and needle with the suture; **c** the ulcer being closed using an endoscopic hand-suturing device; **d** the thread-cutter device stuck on the suture; **e** the second ultrathin scope; **f,g** an electric knife inserted through the second endoscope being used to cut the suture; **h** the ulcer successfully closed with a suture post-treatment.

The double-scope technique is used to overcome difficulties arising from issues when hand-suturing a post-endoscopic mucosal resection ulcer in the duodenum.Video 1

Endoscopy_UCTN_Code_TTT_1AO_2AG

## Notes

Endoscopy E-Videos https://eref.thieme.de/e-videos
*E-Videos* is an open access online section of the journal *Endoscopy* , reporting on interesting cases and new techniques in gastroenterological endoscopy. All papers include a high-quality video and are published with a Creative Commons CC-BY license. Endoscopy E-Videos qualify for HINARI discounts and waivers and eligibility is automatically checked during the submission process. We grant 100% waivers to articles whose corresponding authors are based in Group A countries and 50% waivers to those who are based in Group B countries as classified by Research4Life (see: https://www.research4life.org/access/eligibility/ ). This section has its own submission website at https://mc.manuscriptcentral.com/e-videos .
